# Unique aspects of clinical trials of invasive therapies for chronic pain

**DOI:** 10.1097/PR9.0000000000000687

**Published:** 2018-09-10

**Authors:** Steven P. Cohen, Mark Wallace, Richard L. Rauck, Brett R. Stacey

**Affiliations:** a Department of Anesthesiology, Johns Hopkins School of Medicine, Baltimore, MD, United States; b Department of Anesthesiology, Walter Reed National Military Medical Center, Bethesda, MD, United States; c Division of Pain Medicine, Department of Anesthesiology, University of California San Diego, San Diego, CA, United States; d Center for Clinical Research, Carolinas Pain Institute, Wake Forest University, Winston-Salem, NC, United States; e University of Washington Center for Pain Relief, Department of Anesthesiology, University of Washington, Seattle, WA, United States

**Keywords:** Clinical trial, Design, Invasive, Procedures, Chronic pain

## Abstract

Nearly all who review the literature conclude that the role of invasive procedures to treat chronic pain is poorly characterized because of the lack of “definitive” studies. The overt nature of invasive treatments, along with the risks, technical skills, and costs involved create challenges to study them. However, these challenges do not completely preclude evaluating invasive procedure effectiveness and safety using well-designed methods. This article reviews the challenges of studying outcomes of invasive therapies to treat pain and discuss possible solutions. Although the following discussion can apply to most invasive therapies to treat chronic pain, it is beyond the scope of the article to individually cover every invasive therapy used. Therefore, most of the examples focus on injection therapies to treat spine pain, spinal cord stimulation, and intrathecal drug therapies.

## 1. Introduction

There are a wide range of invasive therapies to treat pain ranging from simple myofascial injections to more invasive and costly procedures such as intrathecal drug therapy. Despite the extensive literature on invasive therapies, there continues to be considerable controversy surrounding efficacy for most of these therapies. For example, among pain management procedures, epidural steroid injections^28^ are the most widely used injections for back pain. Clinical trials evaluating the efficacy of epidural steroid injections vary widely and are heavily influenced by numerous factors such as specialty, with those done by pain physicians more likely to yield positive findings.^23^ It is important to consider biopsychosocial factors when conducting and interpreting clinical studies for pain, and invasive pain therapy trials are not immune to this. Although pain relief can be measured, other important outcomes such as functional capacity, psychological well-being, and return-to-work status can be more difficult to assess.

Invasive therapy trials are more challenging than clinical trials of pharmacologic treatments due to numerous factors. First, there are ethical limitations to consider regarding the use of invasive sham controls that expose patients to risks without benefit and produce additional suffering. Second, there are inherent difficulties in blinding the physician performing the sham-controlled studies, possibly leading to bias. Some treatments unavoidably entail unblinding (conventional spinal cord stimulation), although it may be possible to blind the newer subthreshold stimulation waveforms. Third, the high costs of sham-controlled invasive clinical trials often preclude their conduct because government funding is difficult to obtain for clinical trials involving procedures. Consequently, most clinical trials for invasive therapies have been supported by industries that have a financial interest in the product (eg, neurostimulation and intrathecal drug delivery). This has led to an evidence base for implantable therapies that are replete with positive retrospective and uncontrolled prospective observational studies, as well as randomized trials comparing an invasive therapy with a suboptimal comparator. There is little financial incentive for the industry to sponsor clinical trials evaluating injection therapies, resulting in many small single-site studies that have inadequate power. Fourth, the ability to recruit adequate numbers of participants is extremely challenging, leading to failed studies due to lack of enrollment as well as selection bias that does not necessarily represent the real world.^87^

The current push for more evidence-based medicine has caused the field of invasive therapy pain management to come under intense scrutiny due to lack of evidence for efficacy.^21^ Furthermore, with the prescription drug crisis affecting our country, there is a call for wider use of nonopioid, nonpharmacological, and multimodal therapies to treat pain.^39^ This emphasizes the need for alternative methods besides the standard randomized controlled trial (RCT) to study invasive therapies. Until this happens, invasive pain therapies will continue to experience reduced reimbursement and high scrutiny.

This article will delve into details of the challenges inherent in studying outcomes of invasive therapies to treat pain and discuss possible solutions. Although the following discussion can apply to most invasive therapies to treat chronic pain, it is beyond the scope of the article to individually cover every invasive therapy used. Therefore, most of the examples focus on injection therapies to treat spine pain, spinal cord stimulation, and intrathecal drug therapies.

### 1.1. Epidemiology of injections and surgery

The rise in the number of injections and other interventions has led to increased scrutiny. Although this rise has not been accompanied by concomitant decreases in the prevalence of chronic low back pain, which is the most common and costly chronic pain condition, there is evidence that these may reduce the need for surgery.^40,49,52,86^ Statistics on the rate of use of invasive procedures are limited by the lack of a single, encompassing database. Nevertheless, the rise in interventions is illustrated by data obtained from Medicare records, which show a 107.8% increase in the number of recipients of spinal injections between 2000 and 2008, and a 186.8% increase in the number of procedures performed.^81^ Although epidural steroid injections^28^ comprised a slight majority of procedures, per capita growth rates were higher for facet interventions and sacroiliac joint injections. Similar increases in utilization rates have been shown for spine surgery in general and fusion in particular. Epidemiological studies have found significant geographic variance in the rates of epidural steroid injection,^54^ which cannot be explained by regional differences in health status, and a positive correlation between the rate of injections and the rate of lumbar spine surgery, suggesting that practitioners are not substituting an injection for a more invasive procedure.^36^ Although data are not available regarding the relationship between the number of practitioners who perform injections and the number of procedures performed, there is an almost perfect correlation between the number of spine surgeries and the number of spine surgeons.^25^ The growth in interventions is not uniformly distributed because the highest 10% of pain medicine doctors perform 36.6% of the total number of spinal procedures and 9 times as many as the lowest 10%.^1^

### 1.2. Reasons people fail invasive pain treatments

There are many reasons that people fail invasive pain treatments, with poor patient selection comprising a large majority. Patient-related factors that have been shown to predict poor response to treatment include psychosocial pathology (eg, poorly controlled psychological diagnoses, inadequate coping skills, catastrophizing, substance abuse, and sedentary lifestyle); high levels of clinical disease burden (eg, opioid use, previous failed treatments, high baseline pain scores, and poor function); work-related factors (eg, disability, secondary gain, low job satisfaction, and manual laborer); clinical factors (eg, obesity and old age); and the degree of pathology. Patients with these characteristics are often excluded from clinical trials, and considering how prevalent these variables are in individuals with chronic pain, this raises issues of external validity.

Misdiagnosis is likely the second most common cause of invasive pain treatment failure. For many conditions that are frequently treated with procedures (eg, radiofrequency ablation for facet and sacroiliac joint pain), the diagnosis is predicated on an analgesic response to a low-volume diagnostic injection. These injections have been reported to have false-positive rates ranging between 20% and over 40% based on double blocks using either a saline control injection or a short- and long-acting local anesthetic (whereby longer-lasting relief with the long-acting local anesthetic would constitute a positive response), but the actual false-positive rate is impossible to determine because of the lack of any other means for diagnosis.^10,27,72^ Provocation discography, which purports to identify a painful disc(s) by reproducing one's pain, has also been shown to carry a high false-positive rate in individuals with psychopathology, previous surgery, and other pain complaints.^13,15,118^ The high false-positive rate has resulted in the widely accepted requirement for a negative controlled disc to be obtained during discography and has led some organizations to recommend performing “double blocks” on 2 separate occasions to reduce the false-positive rate before lumbar facet and sacroiliac joint radiofrequency denervation.^9,48^ However, performing multiple blocks will invariably increase the false-negative rate, which will result in the withholding of a safe and effective treatment from individuals who would otherwise benefit. One randomized, comparative cost-effectiveness study found that performing multiple blocks not only reduced the number of overall successful outcomes but also substantially increased costs.^29^

Technical failure accounts for a relatively small proportion of treatment failures and may include the injectate missing the area of pathology during epidural steroid injections; small lesions that miss the target nerve(s) during radiofrequency denervation; lack of pain coverage during spinal cord stimulation; and pseudarthrosis or retained disc fragments during spine surgery. Yet, because it is often remediable, and does not require withholding an effective treatment from someone who might otherwise benefit (ie, a person with multiple risk factors for failure) or performing additional interventions in an attempt to enhance diagnostic specificity (ie, using double blocks to reduce the false-positive rate of a diagnostic injection), a disproportionate volume of literature is devoted to technical improvements. Examples of technical improvements include interventions designed to ensure that an epidural steroid injection reaches the area of pathology (eg, transforaminal approach, contrast injection under fluoroscopy, and use of higher volumes); lesion amplification during radiofrequency denervation (eg, fluid modulation, and higher temperatures and heating time); the use of instrumentation to provide additional stability during spine fusion; and using ultra-high frequency or burst stimulation in preference to conventional stimulation during spinal cord stimulation.^1,22,23,25,32,69,92^

In view of the small effect size for most pain treatments, comparing 2 different treatments or variations of treatments to determine which is best (ie, a comparative-effectiveness study) will generally require many more patients than a placebo-controlled trial, which may itself require hundreds of patients. For many interventions, this is impractical. Yet, there are other ways to establish effectiveness besides RCTs (eg, alternative designs), which was eloquently illustrated by a satirical systematic review published in *BMJ* on the use of parachutes to prevent death or trauma during gravitational challenge, wherein the authors advocated for a “common sense” approach to evaluating evidence for studies involving substantial risk.^101^ For example, if one could show that a selective nerve root block improves the accuracy of diagnosis and accepts as a premise that operating on the correct spinal level will result in better outcomes than performing surgery on the wrong level, then one does not need a clinical trial to demonstrate that selective nerve root blocks could improve surgical outcomes in select patients.^2,38^ Clinical trials and retrospective reviews that have sought to demonstrate a difference in outcomes between 2 treatments (eg, transforaminal vs interlaminar epidural steroid injections or cooled vs conventional radiofrequency denervation) have generally yielded mixed results, which in part can be related to the lack of adequate statistical power and the challenges in controlling for other variables that also affect outcome.^17,23,55,72^

### 1.3. Definition of a successful outcome

Self-reported pain scores (11-point Likert scale or similar visual analogue scale) have typically been used to measure outcome, whether one is evaluating an analgesic drug, an invasive pain procedure, or implanted device (neurostimulator or targeted drug delivery system). Twelve weeks of double-blind conditions is considered the duration of a clinical trial necessary to seek a labeled indication for chronic pain.^44^ However, 12-week trials do not necessarily reflect the risk:benefit and cost:effective analyses for invasive interventions, and all invasive interventions are not the same.

The duration of an invasive trial depends in part on the type of intervention being examined. Generally, interventions that carry high risks and costs are expected to report longer follow-up results. For example, a lumbar spine surgery trial would not be considered relevant or successful if the primary endpoint was measured at 12 weeks; typically, 2 years is considered to be a meaningful outcome period.^116^ A lumbar epidural steroid trial may appropriately use 12 weeks as the primary endpoint to determine success. Radiofrequency denervation trials have traditionally used pain scores at 6 months to measure outcome,^112^ although some randomized trials have used 3 months,^29^ based on patient surveys that suggest that this time frame represents a meaningful result.^29^ Spinal cord stimulation trials have commonly used 6 months as a primary endpoint, but these trials are often extended beyond the initial 6 months to (hopefully) report durable efficacy.^69,76^ Reasons for these extensions are discussed below.

Primary endpoints for most invasive trials continue to be in the form of some type of Likert scale.^42,43,111^ Invasive trials, particularly ones that extend for longer periods, typically include a functional scale such as the Oswestry Disability Index as an important secondary measure. In some studies, these functional scales have been used as either primary endpoints or coprimary endpoints, although coprimary endpoints will increase the required sample size. Authors who advocate functional scales for primary endpoints argue that subjective (11-point) pain scales do not accurately reflect long-term outcomes or provide meaningful results in chronic pain patients.

Reduction in medication use, particularly opioid use, has also been an increasingly relevant outcome in invasive trials. Neuromodulation and other invasive trials^26,29^ have tried to examine this as a potentially important secondary endpoint. Unfortunately, the majority of these trials have yielded disappointing results,^28,57,83,113^ which may in part reflect the observation that the large majority of studies do not have adequate power to detect differences in medication usage. The Kapural et al.^69^ *SENZA* trial reported modest reductions in opioid use, favoring the active arm vs the control arm. In clinical trials seeking a new drug indication, the U.S. Food and Drug Administration (FDA) does not consider an individual outcome as “positive” if the subject reports a meaningful increase in analgesic consumption. For example, most experts would not constitute a 2-point reduction in pain score to be a successful outcome if the study participant doubled his/her opioid dosage and stopped work because of increasing functional impairment.

The duration of an invasive clinical trial correlates rather closely with the cost and risks of the intervention. The costs of an intervention depend on many factors including the anticipated success rate, need for repeat or different additional treatments, likelihood of complications, etc., but in descending order are generally as follows: surgery > neuromodulation (spinal cord stimulation/intrathecal therapy) > minimally invasive intradiscal interventions > radiofrequency denervation > epidural steroid injections > trigger point injections. In the case of spinal cord stimulation and some other interventions, this trend has been driven in part by third-party payers who demand that longer-term outcomes be assessed in clinical trials to justify the expense involved with the intervention.

### 1.4. Sponsorship and other sources of potential bias

It is generally acknowledged that blinding is more important for measuring subjective responses (eg, pain) than for objective ones (eg, vital signs or critical events such as myocardial infarction or death), but the benefits of blinding must be weighed against risks and challenges. In scenarios where blinding of the participant is not possible or feasible, blinding of the outcome assessor can still function to reduce bias.

Not surprisingly, the majority of invasive pain therapy trials have not been double-blinded. Many trials that were conducted over a decade ago were either small RCTs, observational studies with no control arm, or retrospective. Larger, prospective RCTs have become much more commonplace over the past 10 years, particularly in the area of electrical neuromodulation. These larger RCTs have been uniformly sponsored by industry, and many are conducted in compliance with the shift in the FDA's position that in certain contexts, noninferiority studies comparing a new treatment with a proven existing one can be used to establish effectiveness.

Many device RCTs are well-designed with longer durations of follow-up for primary endpoints (6 months) and continued follow-up after the primary endpoint has been reached. However, the concept of industry-sponsored bias, often unintentional, is starting to be questioned by some authors.

Cher and Capobianco^18^ examined the effect of design and sponsorship of device trials for spine surgery. They found 367 trials evaluating spine surgery through www.clinicaltrials.gov, of which 200 (54.5%) were device trials and 167 dealt with other spine-related issues. Device trials were more likely to be sponsored by industry than nondevice trials (74% vs 22%). Multicenter trials were also much more frequently sponsored by industry (80% vs 29%), with approximately 4 times the number of study centers (*P* < 0.0001). The authors found very few multicenter randomized trials in the area of spine surgery that were not sponsored by a device company. The authors concluded, “These findings suggest that previously published studies showing larger effect sizes in industry-sponsored vs non–industry-sponsored studies may be biased as a result of failure to take into account the marked differences in design and purpose.”

Flacco et al.^50^ recently analyzed 319 randomized clinical trials in MEDLINE during 2011 (a random 50% sample) that had over 100 participants. Trials included drugs, biologics, and medical devices evaluated for efficacy and safety. These were head-to-head comparison trials. One hundred eighty-two trials were funded by companies and included 82.3% of the randomized subjects. The majority of trials had only 1 industry sponsor (159/182; 87.3%).

Industry-sponsored trials more commonly used noninferiority/equivalence designs and were cited more frequently. Industry-sponsored trials reported more positive results than non–industry-sponsored trials for superiority or noninferiority/bioequivalence of the experimental arm (odds ratio 2.8; 95% confidence interval: 1.6–4.7). Furthermore, 55 of 57 (96.5%) of noninferiority/bioequivalence trials got favorable ratings. In a later publication, the first author concluded, “The literature of head-to-head RCTs is dominated by industry. Industry-sponsored comparative assessments systematically yield favorable results for the sponsors, even more so when noninferiority designs are involved.”^51^

The previous study should^51^ not be misconstrued or misinterpreted in any way to suggest the industry is falsifying data or providing intentionally misleading information through trial design. Possible explanations for why industry-sponsored trials are more likely than government or nonfunded studies to report positive results include the costs involved for adequately powered studies, more stringent selection criteria, better site selection, and more realistic outcome measures. Several authors (3–7) have demonstrated the many ways in which clinical trials can be unduly biased.^30,47,98,109,114^ Because many device and invasive trials are not double-blinded and using blinded sham procedures can result in ethical concerns raised by institutional review boards, investigators, and industry, at least some degree of bias seems almost inevitable.

Bias can come in many forms. Patients and clinicians often introduce high expectations with device or invasive trials. Whether intentional or not, patients usually understand they have the opportunity to possibly get the “latest and greatest” in treatment, which can amplify the placebo response.^75^ Investigators have to “sell” the clinical trial to prospective enrollees; otherwise, there is often little incentive for patients to participate. Standard texts administered by coordinators, recruiters, and clinicians may be helpful, but subtleties still may produce either positive or negative expectations by the enrollee. The double-blind approach remains the gold standard in all trials when it can be performed ethically and reasonably.

Recently, device and invasive studies have turned to what is often termed “real world” trial design. These may be in the form of observational studies or registries. In the field of pain medicine, these are also most often funded by industry. Some payers have felt these types of designs may be more valuable in evaluating effectiveness than the typical RCT noted above, which is ideal for determining efficacy. Although data can be collected prospectively, many of the previously identified problems still exist, and additional sources of bias are often present because there is no randomized assignment and rarely even a control arm in these trial designs.

### 1.5. Effect of perspective on clinical outcomes

In light of the increased emphasis on evidence-based medicine to inform clinical guidelines and determine reimbursement, a large number of reviews have been published on the most common procedures, such that they now outnumber clinical trial reports by a wide margin. Reviews published by interventional physicians who perform the procedure being evaluated are much more likely to yield positive conclusions and recommendations than those performed by noninterventionalists; this is true for epidural steroid and sacroiliac joint injections, radiofrequency neurotomy, and spine surgery.^20,21,23,27,61,70,78^ These reviews generally use the same methodological quality ratings and grading scales to rate the same clinical studies, which raises the question as to what accounts for such discrepancies. Possible explanations include a better ability of experts who perform a given procedure to evaluate clinical trials; differences in interpretation that reflect differences in the background; and various forms of bias (eg, confirmation, dissemination etc.) on both sides of the debate.^58^

Not only review articles, but clinical trials themselves may be subject to bias in performance, analysis, interpretation, reporting, and publication. One review found that 75% of RCTs evaluating epidural steroid injections in which the corresponding author regularly performed the procedure reached positive conclusions vs 30% in which the corresponding author was a nonpain physician. For review articles, the disparity was larger, with over 90% of those performed by interventional pain physicians concluding epidural steroid injections conferred meaningful benefit vs only about one-third of those performed by nonpain physicians.^23,56^

### 1.6. Technical vs methodological quality

Attempts to explain and reduce these discrepancies have resulted in a recent surge in instruments designed to evaluate the technical quality of invasive clinical trials, just as instruments exist to assess methodological quality.^8,56,79,80^ For facet joint radiofrequency denervation studies, this might include selecting participants based on controlled blocks and ensuring optimal electrode orientation and lesioning parameters.^56^ In studies evaluating surgical interventions, this could include performing preoperative discography and excluding individuals on high-dose opioid therapy and those with concomitant psychopathology before disc replacement or fusion.^77,84^ For epidural steroid injection studies, this might include ensuring that participants presented with true radicular pain confirmed by imaging; optimization of injection technique using contrast injection under fluoroscopic guidance; and exclusion of individuals likely to fail treatment (eg, high-dose opioid users and those with secondary gain) or improve on their own (eg, those with short duration of pain) (12).^8^

### 1.7. Types of clinical trials: what is the best way to study invasive therapies?

Invasive therapy clinical trials are among the most challenging to conduct due to invasiveness/risks, difficulties in blinding, challenges with recruitment, and costs. The type of clinical trial used for invasive therapies will depend on many factors. First and foremost is the underlying question about efficacy or effectiveness. Efficacy is ideally determined through placebo-controlled trials, whereas effectiveness may be determined based on experience using the therapy in clinical practice. A therapy may be efficacious but not effective. Proof of concept (phase IIa) studies are useful to determine the safety of an invasive procedure and to get preliminary evidence of efficacy before embarking on larger and more costly confirmatory clinical trials. Clinical trials designed to determine efficacy evaluate the intervention under carefully controlled situations and emphasize internal validity; however, there are never ideal situations in which to conduct clinical research. These are usually smaller trials that may include sham (performing an invasive procedure at the target site or distant site but no delivery of the therapeutic), placebo (performing the invasive procedure at the target site with delivery of an inactive substance), or active controls. Effectiveness trials are usually larger multisite controlled trials that approach real-world situations, whereas pragmatic trials are conducted in real-world settings; however, even in pragmatic trials, there is some level of control.

The determination of efficacy and effectiveness is a continuum from a single-site randomized placebo-controlled trial to large multicenter pragmatic trials and there is no fine line separating these clinical research methods. Even pragmatic trials may use randomization. Single-site and multisite RCTs can both be used to determine efficacy, whereas multisite studies provide better insight into effectiveness. Observational, comparative-effectiveness, and large pragmatic trials are more geared towards establishing effectiveness in clinical practice settings.^31^ Comparative-effectiveness trials are conducted to determine the relative effectiveness between interventions and can provide information on efficacy when controlled trials separately evaluating each of the interventions exist. An example of a comparative-effectiveness trial is the study of spinal cord stimulation vs reoperation for lumbar radiculopathy. This was a single-site small study performed under carefully controlled conditions in which the results favored spinal cord stimulation.^86^ An attempt was made to perform a similarly large, multicenter comparative-effectiveness trial. After almost 3 years, the study was terminated due to slow enrollment. The major reason was patients' reluctance to be randomized.^87^

Because observational studies often have no comparison groups, they provide limited and potentially biased effectiveness data. Emerging analytic approaches such as the use of propensity score approaches have shown promise in advancing the use of observational data to complement prospective studies. Propensity scoring is a statistical matching technique that attempts to estimate the effect size of a treatment by accounting for the covariates that predict receiving the treatment.^89^ Ultimately, important advantages of well-conducted observational studies include their appreciably lower costs and potentially their ecological or external validity relative to tightly controlled efficacy or even effectiveness studies and pragmatic trials.

Several points should be considered when deciding whether a controlled trial is feasible and necessary, and how to interpret interventional trials. First, lower-risk therapies are usually better candidates for placebo-controlled designs, whereas higher-risk procedures might be better suited for evaluation through comparative-effectiveness, observational, or large pragmatic trial design. It is difficult to justify exposing patients to a high-risk sham procedure without any chance of benefit. This emphasizes the ethics and challenges of determining the efficacy of an invasive therapy before conducting larger comparative-effectiveness trials. For highly invasive therapies, it is not unusual to determine safety first (and get some sense of effectiveness) in observational studies before conducting efficacy trials when possible, or alternatively larger comparative-effectiveness trials.

Second, because invasive therapy trials are often open-label, they are subject to bias. To reduce bias, deviations from standard practice may be required. The decision to deviate from standard practice will depend on the probability of success with the deviation. Therefore, a risk/benefit analysis of the procedure is required and deviations from standard practices with higher risk interventions are acceptable if the potential benefits are high. Examples of this are the ACCELERATE Trial (Clinicaltrials.gov NCT02093793) and the intrathecal gabapentin trial.^93^ The ACCELERATE Trial is comparing 2 methods of stimulation provided by one device. Subjects proceed directly to implantation of a stimulator device without the standard practice of a preimplantation trial. This design is justified and ethical because there is preliminary evidence of success with both methods of stimulation after permanent implant.^69^ As a noninferiority trial design, it is highly likely that some subjects will achieve successful pain relief with one or both of the stimulation methods. The intrathecal gabapentin trial compared 3 doses of gabapentin with placebo. Subjects proceeded directly to an intrathecal drug delivery device (IDDS) implantation without the standard practice of a preimplant drug trial. This was ethically justified because the subjects were allowed to continue to use the IDDS with their drugs of choice after completing the clinical trial, which was characterized by a high success rate. Furthermore, consensus is emerging that preimplantation trials are of limited value and perhaps the standard practice of trialing should be changed.^35^

Third, invasive therapies comparing old and new technologies can be subjected to high placebo (for the new technology) and nocebo (for the old technology) rates. If subjects are not blinded, they are more likely to overestimate the effect of the new technology (ie, it has to be better because it is newer) and underestimate the effect of the old technology (ie, it has to be worse because it is old). This will result in large artificial differences between the 2 therapies that would normalize in real-world conditions. This effect can be confounded even further if the technologies being compared have different sponsors that require management or programming (eg, spinal cord stimulation). This can affect patient-reported outcomes based on technologists' support and motivation (Table 1).

**Table 1:**
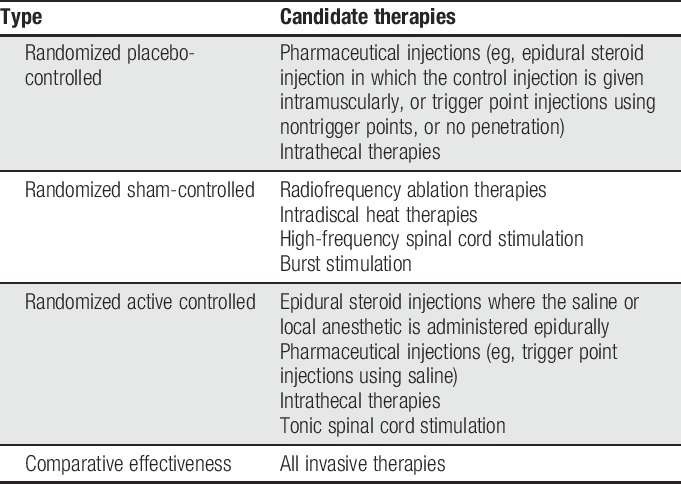
Types of invasive therapy trials with candidate therapies.

The FDA has suggested circumstances in which noninferiority studies can be used to evaluate effectiveness, including for those treatments in which a placebo group would be unethical or impractical (https://www.fda.gov/downloads/Drugs/Guidances/UCM201240.pdf). For certain invasive treatments, such clinical trial designs could be considered. However, there can be major limitations when interpreting these studies, including evaluating efficacy in situations when high-quality placebo-controlled studies have consistently demonstrated efficacy vs a placebo or sham comparator. In such cases, noninferiority could be shown because the trial did not have adequate assay sensitivity, that is, neither active treatment would have shown efficacy vs a placebo or sham treatment if one had been included. For this reason, the results of noninferiority trials that do not include such an inactive comparison group are only interpretable if the treatment being used as the standard has consistently shown superiority vs a placebo or sham treatment, which might not be the case for many types of invasive treatments.

#### 1.7.1. Randomized controlled trials

Randomized controlled trials are among the most challenging trials to conduct for invasive therapies for the reasons discussed above, especially when a placebo or sham arm is involved. Both single-site and multisite RCTs can measure therapeutic efficacy, whereas multisite RCTs provide better information on effectiveness. Both may involve placebo or active controls. Active controls use therapies or agents that have biologic activity that is not expected to have any benefit, although this assumption is not always correct. One example is a study comparing epidural steroids with epidural local anesthetic. Although it is often assumed that epidural local anesthetics do not confer lasting therapeutic benefit when delivered at the same location as the steroids, studies have been shown that they may be comparably effective at short-term follow-up, with both resulting in prolonged pain relief in a small percentage of subjects.^23,104^ Thus, if delivering treatment and “control” agents to the same target, it is best to use a true placebo.

Most patients will not agree to an invasive placebo or sham when there is no chance of benefit. Patients who do agree may not be representative of the real-world population. The acuteness of the pain problem should influence the decision to have a placebo arm. Patients with severe acute pain (such as a lumbar radiculopathy) will unlikely agree to participate in a sham-controlled study, and there are ethical issues that revolve around exposing patients to potential harm without benefit. Furthermore, RCTs that involve placebo or sham controls or “nonstandard of care” treatments require full financial support because no aspects of the study can be billed to insurance. However, RCTs comparing 2 standard-of-care treatments can be billed to most insurance companies. However, there is controversy surrounding these types of trials because the patient population has to have appropriate insurance coverage, thus resulting in bias that may not reflect real-world practices. Allowing for an open-label extension phase after completing the blinded period will increase the chances of recruitment in placebo or sham-controlled studies.

Interventions that are amenable to RCTs with a placebo arm include low-risk therapeutic spine injections such as epidural steroid, facet, and sacroiliac joint injections. It is also possible to perform placebo-controlled RCTs with IDDS therapy as described in the intrathecal gabapentin trial, as well as the early stages of the ziconotide trials.^94,104,115^ However, in the ziconotide trials, ziconotide responders were allowed to continue in an open-label extension period.

The method of placebo or sham delivery in invasive RCTs is highly debatable. Some argue that it is unethical to use placebos with invasive interventions (such as epidural steroid injections) and advocate a less invasive placebo arm such as subcutaneous injection of placebo in the same area as the active intervention. This might provide a better measure of efficacy because evidence has emerged that administration of the “placebo” (such as saline or local anesthetic) onto the actual target site may be similarly therapeutic.^7^ Intrathecal drug delivery device therapy is perhaps the most amenable to an RCT with a placebo arm because the device in place delivers the agents to the same target location without any added risks.

With the exception of spinal cord stimulation, most invasive therapies can use a parallel design or designs that involve switching treatments for those patients who do not respond to the initial treatment. There are several considerations in determining which design to use. First, one must determine whether there exists a possible carry-over effect. For example, steroid injections can have prolonged effects that may carry over to a subsequent period. In such circumstances, a standard cross-over design in which patients are randomized to receive 2 or more treatments in different order cannot be used. However, it is also possible to have subjects switched to the other treatment if they do not achieve pain relief from the first treatment they are randomized to. This design was used in a study comparing spinal cord stimulation with reoperation for failed back surgery to treat low back and leg pain. Patients were randomized to either arm and only switched treatments if the first treatment was unsuccessful.^86^ Second, one must decide whether it is possible to blind the therapies. It is possible to blind the patient for IDDS therapies and most spinal injection therapies if the technique used between treatment periods with different interventions is similar. However, it is not possible to blind most spinal cord stimulation studies because the subjects can feel whether or not they are receiving stimulation. An exception to this is high-frequency stimulation, in which the stimulation is subperception threshold. However, there are currently no studies comparing active and sham high-frequency stimulation. Furthermore, high-frequency stimulation results in rapid battery consumption, which could possibly unblind the therapy unless precautions are taken. Unless the placebo arm technique is the same as the active arm (eg, epidural steroids vs epidural saline), blinding the interventionalist is not possible. In addition, if the 2 techniques are drastically different (ie, epidural vs subcutaneous injection), it may be difficult to blind the patient. There are currently 2 randomized, controlled cross-over spinal cord stimulator trials in progress (SUNBURST and ACCELERATE, clinicaltrials.gov). The advantage of such a design is much greater power for the between-group comparison. However, the subjects are unblinded because the stimulation feels different between the 2 methods (Tonic vs Burst; Tonic vs High Frequency).

Invasive therapy trials are also amenable to adaptive trial design. Traditional RCTs contain prespecified patient-reported outcome measures and calculate subject enrollment based on a power analysis. Adaptive trials allow for the adjustment of certain aspects of the trial design midstream based on accumulating data on efficacy and safety.^6^ Given the invasiveness and increased risks associated with invasive therapies, it is reasonable to consider interim analyses at predefined milestones in the trial and adjust plans accordingly by dropping arms or stopping the study altogether in response to compelling early evidence of efficacy, futility, or harm.

#### 1.7.2. Comparative effectiveness

In 2008, The American Reinvestment and Recovery Act allocated over $1 billion to support comparative effectiveness research (CER).^16^ In a 2009 report by the Institute of Medicine, CER was defined as the generation and synthesis of evidence that compares the benefits and harms of alternative methods to prevent, diagnose, treat, and monitor a clinical condition or improve the delivery of care.^103^

Given the challenges of conducting placebo-controlled RCTs with invasive therapies, CER seems to be an important and effective alternative. Unlike RCTs, CER aims to represent real-world practice. However, an obstacle in conducting CER for invasive therapies is the lack of data on efficacy and effectiveness. As mentioned earlier in this article, one needs some evidence of efficacy before embarking on comparisons of different treatments that do not include a placebo or sham control. The only way to provide compelling evidence of this is to conduct placebo or sham-controlled trials, which are often lacking in invasive pain therapies. There are various methods of conducting CER.

#### 1.7.3. Observational studies

Observational studies use real-world patient care data and outcomes to determine things such as therapy effectiveness, patient selection, cost-effectiveness, safety, and the effect of comorbidities on outcome. Data sources are typically medical records, surveys, registries, or insurance databases. Observational studies have significant limitations because they require very large numbers of patients to reach meaningful conclusions and they require some effective means of reducing confounding factors, either by design or through sophisticated analytical methods. An advantage is that they include real-world patient data that may be more meaningful to providers and insurance carriers. A disadvantage is the requirement for cooperation among busy clinicians to collect and enter the data (although much of the data can be entered by patients), thereby jeopardizing data quality.^102^ There have been several attempts to create pain registries, which have been slowed due to cost and time constraints. Electronic medical records (EMRs) have the potential to generate a large amount of patient care data on a variety of therapies. However, this will require EMR systems capable of communicating and overcoming the HIPAA barriers. Large health care systems that have the same EMR, such as the Veterans Health Administration, currently have the potential to contribute data to observational studies. However, single health care systems may not translate into the wide range of health care environments across the country.

Registries can be disease-based, patient-based, or treatment-based, with treatment-based being most applicable for procedural interventions. One example of an invasive therapy observational study might involve researchers who want to evaluate the effects of a single spinal cord stimulation technology on low back and leg pain. They recruit 20 sites around the country that implant the device. Using a prespecified list of demographics and clinical information, they access medical records to extract the data over time including preimplant and postimplant. Data can be extracted retrospectively, but patients are followed prospectively over time. The FDA has suggested using historical controls to tease out the effects of treatment vs natural course in scenarios when placebo-controlled trials are not indicated (https://www.fda.gov/downloads/Drugs/Guidances/UCM201240.pdf). However, temporal changes in placebo group responses and effect sizes in analgesic clinical trials render this approach highly problematic when evaluating treatments for chronic pain.

#### 1.7.4. Pragmatic trials

Pragmatic trials use multiple sites to randomize large numbers of typical patients in real-world typical practice settings. This is in contrast to RCTs that study patients under ideal circumstances and have strict inclusion and exclusion criteria. Pragmatic trials represent an amalgam of randomized and observational studies, containing both components.^110^ These trials randomize patients to treatments and can obtain useful data on large numbers of patients.

Pragmatic trials have been used to study treatments in fields such as cardiology and oncology, but have not been extensively used for invasive therapies to treat chronic pain. However, given the challenges and complexities inherent in conducting RCTs for invasive therapies, pragmatic trials would likely be very valuable because patients would receive therapy more in the comfort of their real-world setting. Although it is easier to recruit subjects, the results can sometimes be challenging to interpret.

An example of a pragmatic trial is the Spine Patient Outcomes Research Trial (SPORT), which was a 5-year study that compared surgical and nonsurgical treatments for 3 of the most common back conditions. Approximately 2500 patients took part in the study, which was conducted at 13 sites across the United States. After watching a shared decision-making video that described what is known about back surgery vs nonoperative care, patients who agreed to participate were randomized between treatments. Both treatments were part of standard of care and sites were allowed to provide treatments based on standard practices. An observational group consisted of those who elected not to participate in randomization and choose their own treatment approach, but agreed to be followed for outcomes over the ensuing 12 to 48 months.^117^ This approach could be applied to a number of invasive therapies; for example, randomizing patients to either epidural steroid injections or physical therapy for acute lumbar radiculopathy.

Because these trials have limited funding and involve large numbers of patients at multiple sites, only the most pertinent data are collected. This is a shortcoming because important data that could be used for decision-making may be missing. Another confounding factor is the challenge insurance coverage plays. Many invasive therapies for chronic pain are limited by lack of insurance authorization. A solution for this is to create a third observational group consisting of patients who cannot receive the therapy due to insurance coverage, but agree to be followed for outcomes. However, this approach introduces the confounding issue of lack of randomization.

#### 1.7.5. Cluster randomized trials

Intrathecal drug delivery device therapy and spinal cord stimulation involve the use of various devices with different technologies and manufacturers. In real-world practice, clinics usually choose one device to use for their patient population. This practice is driven by comfort with one technology, complexities of dealing with multiple manufacturers and technician support, less confusion for patients on choices, and an administrative desire to obtain the best contracts for cost savings. However, little is known about the comparative effectiveness of these different devices. Cluster randomized trial design may be ideally suited for studies intended to compare different devices and treatment regimens. In cluster randomized trials, randomization is by group, such as those treated at a particular clinic site, rather than by individual patient. Each site implements only one intervention. This trial design mimics real-world practice and can effectively compare various devices.^91^

#### 1.7.6. Single-patient (n-of-1) trials

As discussed above, it is very challenging to conduct standard parallel-group RCTs to evaluate invasive therapies. There challenges also extend to the design, execution, and interpretation of observational and large pragmatic studies. It has been suggested that single-patient (N-of-1) trials have the potential to answer questions about effectiveness in disease states with high patient variability (ie, 1 size does not fit all). N-of-1 trials are multiple-period cross-over experiments comparing 2 or more treatments within individual patients. Factors favoring the use of these types of trials include heterogeneity of treatment effects, chronicity, stability, negligible carry-over effects of the therapies, and lack of adequate evidence.^41^ Chronic pain conditions can meet all these requirements, making them potentially amenable to N-of-1 studies. The invasive therapies that are most conducive to N-of-1 studies are spinal cord stimulation and IDDS. Spinal cord stimulation technology is exploding, with a plethora of current and emerging stimulation parameters. In addition, there are a number of different agents, in various combinations, being used in IDDS. N-of-1 studies seem to be a promising vehicle to evaluate the effectiveness of these various interventions.

### 1.8. Challenges in blinding invasive therapy studies

Systematic reviews evaluating double-blind, placebo-controlled studies, and the studies themselves represent the highest level of evidence in medicine (Centre for Evidence-Based Medicine, Levels of Evidence, Oxford University, UK. Available at: https://www.essentialevidenceplus.com/product/ebm_loe.cfm?show=oxford). Randomized controlled trials comparing active and placebo medications in a double-blind fashion for pain conditions such as headache have been conducted for over 50 years, and have become the reference standard by which regulatory agencies, 3rd party payers, and organizations that prepare and distribute guidelines determine efficacy. However, the use of clinical trials to evaluate procedural interventions did not become routine until much later. For example, the first randomized trial evaluating an invasive pain treatment was in 1970, when Swerdlow and Sayle-Creer compared epidural steroid with epidural saline and epidural local anesthetic in a nonblinded fashion.^106^

Although using a placebo control in a blinded fashion is necessary to determine efficacy, effectiveness can often be determined by other means, such as large, pragmatic clinical trials and registries. Yet, nonblinded trials may overestimate the effect size. Systematic reviews and clinical trials have estimated that a lack of blinding exaggerates the treatment effect by approximately 35%, and that unclear blinding is associated with increases in the effect estimate by 13% to 25%.^60,97,119^

There are inherent challenges to blinding clinical trials evaluating procedures, which are commensurate with the invasiveness of the intervention. Hence, it is not always possible, or practical, to evaluate invasive procedures in a double-blind fashion. In a negative study evaluating arthroscopic knee surgery for osteoarthritis, Moseley et al.^85^ performed a patient- and evaluator-blinded 3-arm parallel study comparing arthroscopic debridement, arthroscopic lavage, and a sham procedure. Those in the first 2 groups underwent general anesthesia, whereas those who received a sham procedure received intravenous sedation and a 1-cm incision without scope insertion. By exposing patients to the risks of sedation and a surgical incision, this study generated a great deal of controversy.^59^ A similar negative study performed several years later that was also published in the *New England Journal of Medicine* blinded only the evaluator, by placing a neoprene sleeve over the affected knee at follow-up.^73^ The value of blinding an evaluator when the patient is not blinded and the primary outcome is based on subjective self-report is unclear.

Studies evaluating intradiscal procedures for discogenic low back pain, which carry a risk of discitis, a condition with no reliably effective treatment options, have encountered similar obstacles in trying to balance the benefits and risks of performing a double-blind study. There are 2 double-blind studies evaluating intradiscal electrothermal therapy and one evaluating biacuplasty.^53,68,88^ The earliest study that assessed intradiscal heating was performed by an interventional pain medicine group and demonstrated modest benefit at 6-month follow-up.^88^ However, a subsequent randomized controlled study conducted by spine surgeons that failed to recruit enough patients based on their prestudy power analysis yielded no patients in either group who experienced a positive categorical outcome.^53^ In the earlier study, a large introducer was placed into the disc but the electrode was not inserted, whereas the negative second study involved the insertion of both the introducer and electrode.^53,88^ More recently, a large, prospective case-control study found that penetrating an intervertebral disc during discography, even with a much smaller needle than an intradiscal electrothermal cautery introducer, may accelerate disc degeneration and increase the risk of disc herniation,^14^ which is consistent with animal studies that use annular puncture as a model for disc degeneration.^71^ This raises concerns regarding the risks participants are exposed to during double-blind, intradiscal studies. In addition to issues regarding the ethics of intradiscal sham treatments, questions have been raised about the effectiveness of blinding these studies because heating a disc for over 15 minutes is more painful than sham ablation.

There have been well over 40 placebo-controlled trials evaluating epidural steroid injections, the most commonly performed procedure in pain clinics throughout the world.^23,81^ A majority of these studies have used epidural saline or local anesthetic injections as the placebo group, rather than intramuscular injections which are used less frequently. One major advantage of using an epidural nonsteroid injection as the control compared with an intramuscular injection is that the patient experience is the same, which promotes effective blinding. This is particularly relevant for transforaminal epidural injections, which frequently reproduce a patient's radicular pain. However, a recent systematic review and meta-analysis found that epidural nonsteroid injections are not a true placebo; in fact, the authors concluded that more than half of the early therapeutic effect of an epidural injection is due to the injection itself, rather than the steroid.^7^

An intrathecal study evaluating the snail toxin ziconotide for cancer and AIDS-related pain administered the medication or a saline placebo for up to 10 days before nonresponders could cross-over. Although over two-thirds of the control group experienced at least one adverse event, only 5% reported an injection site reaction and the incidence of headache was similar for both groups, which suggest that blinding in some invasive trials can be successfully and safely implemented.^104^

Facet joint interventions comprise the 2nd most frequently performed procedure in pain management centers, and there have been over half a dozen placebo-controlled studies evaluating radiofrequency denervation. Radiofrequency ablation, which involves lesioning small, pain-transmitting nerves using the application of heat generated from alternating current, has been used in RCTs not only for facet joint pain but also for sacroiliac joint pain and knee osteoarthritis.^19,24^ As alluded to for intradiscal electrothermal therapy, blinding can be difficult when a treatment involves heat ablation, which is typically perceived as painful. Therefore, the injection of local anesthetic before radiofrequency ablation to reduce procedure-related pain has become standard of care in clinical practice. Preclinical studies have also demonstrated that the preadministration of lidocaine before denervation enhances the lesion size, and may, therefore, reduce the risk of technical failure.^92^ In all high-quality clinical trials evaluating the efficacy of radiofrequency denervation, the control group received local anesthetic administration before sham denervation was applied to the medial branches (for facet joint pain), lateral branches (for sacroiliac joint pain), or the genicular nerves (for knee osteoarthritis), which enabled effective blinding.^27^ Some investigators also added corticosteroid to the local anesthetic,^29^ which was shown in one randomized study to reduce the incidence of postablation neuritis.^37^ But, the preinjection of local anesthetic, with or without steroids, may increase the response rate in the sham group, because some randomized studies have found that a significant proportion of patients may experience prolonged pain relief with nerve blocks performed with local anesthetic.^82^ Inadvertent facet blocks performed to reduce procedure-related pain in the treatment group in placebo-controlled trials evaluating vertebroplasty have been suggested by many experts to account for the failure to demonstrate significant benefit.^12,64^

These factors raise a dilemma for researchers designing clinical trials: Should one use a true placebo and risk undermining the effectiveness of blinding, or try to replicate the experience of the treatment group, in which case the control treatment may confer an analgesic benefit? Similar to epidural injections and nerve blocks, studies have also found that intra-articular saline may provide pain relief and functional improvement.^45^

### 1.9. Placebo effect

Nonspecific placebo effects (a measurable clinical response to a presumed inactive substance, procedure, or interaction) have long been recognized in the field of pain medicine. Therefore, the “placebo-controlled trial” is the reference standard to determine the “true effect” of treatments offered to patients.^4^ An alternative definition, “improvements in patients' symptoms that are attributable to their participation in the therapeutic encounter, with its rituals, symbols, and interactions,” emphasizes that the effect is much more than the treatment. It involves context, meaning, situation, expectation, and a host of other factors.^66^ These improvements are real, frequently partial, and likely add to the overall “effectiveness” of the clinical encounter, meaning there is clinical benefit to the placebo response. In practice, clinicians cannot distinguish what proportion of a positive response to treatment is attributable to the placebo effect, nor is it important to do so.

The magnitude of the analgesic placebo effect is variable from less than 10% to in excess of 50% and may be of greater magnitude than the “true” treatment effect, obscuring the effect of an active treatment in the context of a clinical trial. This is evidenced by the fact that in nearly all controlled analgesic drug and interventional trials, the within-group difference for the “control” group is greater than the between-group difference for pain scores.^28,53,55,68,88^ In general, a goal of a well-conducted trial is to minimize the placebo response to maximize the chance of detecting a true treatment effect. The response also varies with the type of pain. For example, the placebo effect in neuropathic pain clinical trials varies by diagnosis.^3,33^ The understanding of the neurobiology of the placebo response is evolving, but it is clear that the response is not static, and a specific patient's response to a placebo is variable and potentially modifiable.^5,34,62,65,67,74,90,95,99,100,105,108^ Importantly, for interventional pain medicine practitioners, invasive “sham” procedures and devices have a higher placebo response than noninvasive controls.^63,65,67,74^

As interventions for pain treatment invariably occur in the clinical setting as a "therapeutic encounter", the placebo response is common and some argue that it is enhanced.^11,46,96,107^ For clinical trials evaluating these procedures, minimizing the placebo response is one way of increasing assay sensitivity (ie, increasing the likelihood of detecting a real treatment effect).

There is also ambiguity as to what “constitutes” a true placebo and whether true placebo arms are even possible when evaluating certain procedures. For epidural steroid injections, a systematic review and meta-analysis found that randomized trials in which epidural nonsteroid solutions were considered to be the control injection were more likely to be negative than those in which nonepidural (eg, intramuscular) injections were used as the control, suggesting epidural saline and local anesthetic have therapeutic value.^7^ For certain interventions such as those involving thermal lesioning (eg, biacuplasty) or conventional spinal cord stimulation, a true placebo-controlled trial may not even be possible.

Invasive pain therapy procedures and sham devices have risks associated with them because procedures are inherently invasive. In the example provided previously, for intradiscal procedures, even entering the disc may be associated with an increased rate of disc degeneration and herniation.^14^ Are these risks justified or outweighed by the interest of science or by the potential clinical benefit of a placebo response?^11^ Most placebo-controlled studies evaluate medications and are funded by the pharmaceutical industry. Because funding for expensive invasive pain procedures is challenging, who bears the cost of a placebo procedure, or of complications related to it? Are thoughtfully designed, randomized, large pragmatic comparative-effectiveness (not necessarily sham/placebo) trials adequate to obtain the scientific information necessary to validate or reject invasive treatments? Because the response to procedures is variable, it is possible that focusing on phenotyping and responder analysis in large, pragmatic studies may provide additional clinically useful information. In fact, the NIH task force on research in pain medicine has identified registries, which can aid in phenotyping and responder analysis, as a top-ten research priority. Yet, despite the risks, costs, and ethics of using sham controls in invasive pain therapy studies, there are numerous studies that have used sham controls.

## 2. Conclusions

Chronic pain is a major drain on our economy and will remain a major societal burden for the foreseeable future. Although there are myriad potential pain treatments available, the data that should enable us to wisely select between them in specific patient populations are lacking. This is particularly true for invasive therapy options in pain care, where the additional complexities of technical details, expectations, and risks make evaluating their role even more challenging. As a result, nearly all who review the literature conclude that the role for interventions is not well defined because of the lack of “definitive” studies.

It is not lack of clinical, economic, or academic work in the area of invasive pain therapies that leads to this lack of data; rather, as reviewed in this article, numerous barriers to definitive RCTs assessing invasive pain therapies have limited the available literature. Practically, this means that clinicians, regulators, payers, and patients are faced with making treatment choices in difficult situations with less-than-ideal information at hand. Although all clamor for the rarely reported objective RCT with blinding and a “placebo” or sham arm, the lack of such studies mean real-world decisions are made with lower-quality evidence. Comparative-effectiveness research offers one potential path forward, but interpreting them can be difficult. The challenge for researchers of invasive pain therapies and funders of such research is therefore to conduct smaller scale placebo- or sham-controlled trials from which estimates for efficacy and effectiveness are derived, which in turn can be used to build meaningful CER.

Safely and effectively performing procedures in the context of a study requires an interested, invested, skilled, experienced, and involved provider with knowledge of each individual patient's anatomy and physiology. Importantly, research on these pain procedures must spring from the minds and practices of those who perform the procedures and apply academic rigor to their work. The conduct and design of any given study cannot be removed from the clinical realm, lest it be “well-designed” only in the abstract.

The overt nature of invasive treatments, along with the risks, technical skills, and costs, will always remain barriers to studying them (Table 2). Involvement of nonproceduralists to assure that all domains and aspects of care are addressed is essential for external validity. Separation of those who perform the procedure from those who evaluate the outcomes of the procedure reduces bias. Recognition of the barriers to standards that apply to medication trials (placebo-controlled, double-blind), as applied to procedures, is important for those who critique studies to bear in mind. Recruitment for studies that involve “the latest and greatest” vs anything else will continue to remain a challenge, as will managing the nocebo and placebo effects in these studies.

**Table 2:**
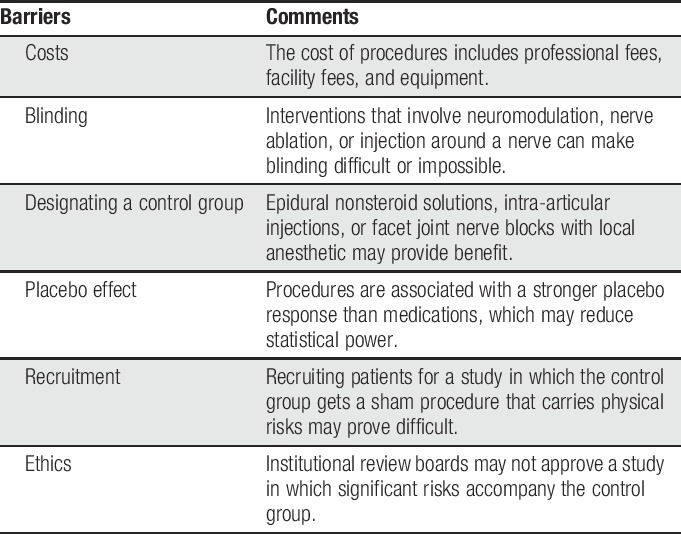
Major barriers to performing randomized, invasive therapy trials for chronic pain conditions.

The obstacles to high-quality studies are real but potentially surmountable. Recent publications reveal progress with more robust design of invasive therapy studies—larger sample sizes, better characterization of subjects, randomized designs, varied outcome measures, and longer-term data collection.

Finally, although funding is a barrier to all clinical research, it is a major hurdle for expensive and potentially risky procedures and pain devices. Industry funding is prevalent but raises concerns about the possibility of various types of bias. Insurance companies do not want to pay for less effective or unproven treatments, or for “control” arms. It is difficult to find enthusiasm for funding the second, “confirmatory” study that is necessary to understand or confirm the true effect of a procedure. Thus, alternative sources for funding, pooling of resources, and creativity are required to find the means to study pain procedures more systematically. This is a challenge not just for those who perform these procedures, but considering the stakes, the medical community in general.

## Disclosures

The authors whose names are listed immediately below report the following details of affiliation or involvement in an organization or entity with a financial or nonfinancial interest in the subject matter or materials discussed in this article.

S.P. Cohen reports other from Halyard, other from SPR Medical, other from Medtronic, other from Abbott, and other from Zynerba, during the conduct of the study; M. Wallace reports other from Boston Scientific and other from Jazz Pharmaceutical, outside the submitted work; R.L. Rauck reports other from Pfizer, personal fees and other from BioDelivery Sciences, Inc, (BDSI), personal fees and other from Boston Scientific, other from Mainstay, other from Saluda, other from SPR Therapeutics, other from Neuros, personal fees and other from Insys, personal fees from Daiichi Sankyo, personal fees and other from Jazz Pharmaceutical, other from Stimwave, other from Vertos, personal fees and other from Egalet, personal fees and other from Medtronic, and other from Grunenthal, outside the submitted work; B.R. Stacey reports grants from Pfizer, other from Kineta, grants from Axsome, grants from Teva, and grants from Vertex, outside the submitted work.

## References

[1] Abbott ZI Nair KV Allen RR Akuthota VR Utilization characteristics of spinal interventions. Spine J 2012;12:35–43. 22138113 10.1016/j.spinee.2011.10.005

[2] Anderberg L Annertz M Brandt L Saveland H Selective diagnostic cervical nerve root block–correlation with clinical symptoms and MRI-pathology. Acta Neurochir (Wien) 2004;146:559–65; discussion 565. 15168223 10.1007/s00701-004-0241-4

[3] Arakawa A Kaneko M Narukawa M An investigation of factors contributing to higher levels of placebo response in clinical trials in neuropathic pain: a systematic review and meta-analysis. Clin Drug Investig 2015;35:67–81. 10.1007/s40261-014-0259-1 25559268

[4] Benedetti F Beecher as clinical investigator: pain and the placebo effect. Perspect Biol Med 2016;59:37–45. 27499483 10.1353/pbm.2016.0022

[5] Benedetti F Dogue S Different placebos, different mechanisms, different outcomes: lessons for clinical trials. PLoS One 2015;10:e0140967. 26536471 10.1371/journal.pone.0140967 PMC4633056

[6] Berry DA Bayesian clinical trials. Nat Rev Drug Discov 2006;5:27–36. 16485344 10.1038/nrd1927

[7] Bicket MC Gupta A Brown CHt Cohen SP Epidural injections for spinal pain: a systematic review and meta-analysis evaluating the “control” injections in randomized controlled trials. Anesthesiology 2013;119:907–31. 24195874 10.1097/ALN.0b013e31829c2ddd

[8] Bicket MC Hurley RW Moon JY Brummett CM Hanling S Huntoon MA van Zundert J Cohen SP The development and validation of a quality assessment and rating of technique for injections of the spine (AQUARIUS). Reg Anesth Pain Med 2016;41:80–5. 26655218 10.1097/AAP.0000000000000337

[9] Bogduk N International Spinal Injection Society guidelines for the performance of spinal injection procedures. Part 1: zygapophysial joint blocks. Clin J Pain 1997;13:285–302. 9430809 10.1097/00002508-199712000-00003

[10] Boswell MV Manchikanti L Kaye AD Bakshi S Gharibo CG Gupta S Jha SS Nampiaparampil DE Simopoulos TT Hirsch JA A best-evidence systematic appraisal of the diagnostic accuracy and utility of facet (zygapophysial) joint injections in chronic spinal pain. Pain Physician 2015;18:E497–533. 26218947

[11] Brim RL Miller FG The potential benefit of the placebo effect in sham-controlled trials: implications for risk-benefit assessments and informed consent. J Med Ethics 2013;39:703–7. 23239742 10.1136/medethics-2012-101045 PMC3812890

[12] Buchbinder R Osborne RH Ebeling PR Wark JD Mitchell P Wriedt C Graves S Staples MP Murphy B A randomized trial of vertebroplasty for painful osteoporotic vertebral fractures. N Engl J Med 2009;361:557–68. 19657121 10.1056/NEJMoa0900429

[13] Carragee EJ Chen Y Tanner CM Truong T Lau E Brito JL Provocative discography in patients after limited lumbar discectomy: a controlled, randomized study of pain response in symptomatic and asymptomatic subjects. Spine (Phila Pa 1976) 2000;25:3065–71. 11145818 10.1097/00007632-200012010-00014

[14] Carragee EJ Don AS Hurwitz EL Cuellar JM Carrino JA Herzog R 2009 ISSLS Prize Winner: does discography cause accelerated progression of degeneration changes in the lumbar disc: a ten-year matched cohort study. Spine (Phila Pa 1976) 2009;34:2338–45. 19755936 10.1097/BRS.0b013e3181ab5432

[15] Carragee EJ Tanner CM Khurana S Hayward C Welsh J Date E Truong T Rossi M Hagle C The rates of false-positive lumbar discography in select patients without low back symptoms. Spine (Phila Pa 1976) 2000;25:1373–80; discussion 1381. 10828919 10.1097/00007632-200006010-00009

[16] Cervero F Gilbert R Hammond RG Tanner J Development of secondary hyperalgesia following non-painful thermal stimulation of the skin: a psychophysical study in man. PAIN 1993;54:181–9. 8233532 10.1016/0304-3959(93)90207-6

[17] Cheng J Pope JE Dalton JE Cheng O Bensitel A Comparative outcomes of cooled versus traditional radiofrequency ablation of the lateral branches for sacroiliac joint pain. Clin J Pain 2013;29:132–7. 22688606 10.1097/AJP.0b013e3182490a17

[18] Cher DJ Capobianco RA Spine device clinical trials: design and sponsorship. Spine J 2015;15:1133–40. 25637470 10.1016/j.spinee.2015.01.027

[19] Choi WJ Hwang SJ Song JG Leem JG Kang YU Park PH Shin JW Radiofrequency treatment relieves chronic knee osteoarthritis pain: a double-blind randomized controlled trial. PAIN 2011;152:481–7. 21055873 10.1016/j.pain.2010.09.029

[20] Chou R Atlas SJ Stanos SP Rosenquist RW Nonsurgical interventional therapies for low back pain: a review of the evidence for an American Pain Society clinical practice guideline. Spine (Phila Pa 1976) 2009;34:1078–93. 19363456 10.1097/BRS.0b013e3181a103b1

[21] Chou R Hashimoto R Friedly J Fu R Bougatsos C Dana T Sullivan SD Jarvik J Epidural corticosteroid injections for radiculopathy and spinal stenosis: a systematic review and meta-analysis. Ann Intern Med 2015;163:373–81. 26302454 10.7326/M15-0934

[22] Cohen SP Argoff CE Carragee EJ Management of low back pain. BMJ 2008;337:a2718. 19103627 10.1136/bmj.a2718

[23] Cohen SP Bicket MC Jamison D Wilkinson I Rathmell JP Epidural steroids: a comprehensive, evidence-based review. Reg Anesth Pain Med 2013;38:175–200. 23598728 10.1097/AAP.0b013e31828ea086

[24] Cohen SP Chen Y Neufeld NJ Sacroiliac joint pain: a comprehensive review of epidemiology, diagnosis and treatment. Expert Rev Neurother 2013;13:99–116. 23253394 10.1586/ern.12.148

[25] Cohen SP Deyo RA A call to arms: the credibility gap in interventional pain medicine and recommendations for future research. Pain Med 2013;14:1280–3. 23819693 10.1111/pme.12186

[26] Cohen SP Hanling S Bicket MC White RL Veizi E Kurihara C Zhao Z Hayek S Guthmiller KB Griffith SR Gordin V White MA Vorobeychik Y Pasquina PF Epidural steroid injections compared with gabapentin for lumbosacral radicular pain: multicenter randomized double blind comparative efficacy study. BMJ 2015;350:h1748. 25883095 10.1136/bmj.h1748 PMC4410617

[27] Cohen SP Huang JH Brummett C Facet joint pain–advances in patient selection and treatment. Nat Rev Rheumatol 2013;9:101–16. 23165358 10.1038/nrrheum.2012.198

[28] Cohen SP White RL Kurihara C Larkin TM Chang A Griffith SR Gilligan C Larkin R Morlando B Pasquina PF Yaksh TL Nguyen C Epidural steroids, etanercept, or saline in subacute sciatica: a multicenter, randomized trial. Ann Intern Med 2012;156:551–9. 22508732 10.7326/0003-4819-156-8-201204170-00397

[29] Cohen SP Williams KA Kurihara C Nguyen C Shields C Kim P Griffith SR Larkin TM Crooks M Williams N Morlando B Strassels SA Multicenter, randomized, comparative cost-effectiveness study comparing 0, 1, and 2 diagnostic medial branch (facet joint nerve) block treatment paradigms before lumbar facet radiofrequency denervation. Anesthesiology 2010;113:395–405. 20613471 10.1097/ALN.0b013e3181e33ae5

[30] Colagiuri B Participant expectancies in double-blind randomized placebo-controlled trials: potential limitations to trial validity. Clin Trials 2010;7:246–55. 20421243 10.1177/1740774510367916

[31] Concato J Shah N Horwitz RI Randomized, controlled trials, observational studies, and the hierarchy of research designs. N Engl J Med 2000;342:1887–92. 10861325 10.1056/NEJM200006223422507 PMC1557642

[32] Cosman ER Jr Dolensky JR Hoffman RA Factors that affect radiofrequency heat lesion size. Pain Med 2014;15:2020–36. 25312825 10.1111/pme.12566

[33] Cragg JJ Warner FM Finnerup NB Jensen MP Mercier C Richards JS Wrigley P Soler D Kramer JL Meta-analysis of placebo responses in central neuropathic pain: impact of subject, study, and pain characteristics. PAIN 2016;157:530–40. 26588698 10.1097/j.pain.0000000000000431

[34] Czerniak E Biegon A Ziv A Karnieli-Miller O Weiser M Alon U Citron A Manipulating the placebo response in experimental pain by altering doctor's performance style. Front Psychol 2016;7:874. 27445878 10.3389/fpsyg.2016.00874 PMC4928147

[35] Deer TR Prager J Levy R Burton A Buchser E Caraway D Cousins M De Andres J Diwan S Erdek M Grigsby E Huntoon M Jacobs M Kim P Kumar K Leong M Liem L McDowell G Panchal SJ Rauck R Saulino M Staats P Stanton-Hicks M Stearns L Sitzman BT Wallace M Willis KD Witt W Yaksh T Mekhail N Polyanalgesic Consensus Conference–2012: recommendations on trialing for intrathecal (intraspinal) drug delivery: report of an interdisciplinary expert panel. Neuromodulation 2012;15:420–35; discussion 435. 22494357 10.1111/j.1525-1403.2012.00450.x

[36] Deyo RA Mirza SK Martin BI Back pain prevalence and visit rates: estimates from U.S. national surveys, 2002. Spine (Phila Pa 1976) 2006;31:2724–7. 17077742 10.1097/01.brs.0000244618.06877.cd

[37] Dobrogowski J Wrzosek A Wordliczek J Radiofrequency denervation with or without addition of pentoxifylline or methylprednisolone for chronic lumbar zygapophysial joint pain. Pharmacol Rep 2005;57:475–80. 16129914

[38] Dooley JF McBroom RJ Taguchi T Macnab I Nerve root infiltration in the diagnosis of radicular pain. Spine (Phila Pa 1976) 1988;13:79–83. 2967996 10.1097/00007632-198801000-00019

[39] Dowell D Haegerich TM Chou R CDC guideline for prescribing opioids for chronic pain–United States, 2016. JAMA 2016;315:1624–45. 26977696 10.1001/jama.2016.1464 PMC6390846

[40] Driscoll T Jacklyn G Orchard J Passmore E Vos T Freedman G Lim S Punnett L The global burden of occupationally related low back pain: estimates from the Global Burden of Disease 2010 study. Ann Rheum Dis 2014;73:975–81. 24665117 10.1136/annrheumdis-2013-204631

[41] Duan N Kravitz RL Schmid CH Single-patient (n-of-1) trials: a pragmatic clinical decision methodology for patient-centered comparative effectiveness research. J Clin Epidemiol 2013;66:S21–8. 23849149 10.1016/j.jclinepi.2013.04.006 PMC3972259

[42] Dworkin RH Turk DC Farrar JT Haythornthwaite JA Jensen MP Katz NP Kerns RD Stucki G Allen RR Bellamy N Carr DB Chandler J Cowan P Dionne R Galer BS Hertz S Jadad AR Kramer LD Manning DC Martin S McCormick CG McDermott MP McGrath P Quessy S Rappaport BA Robbins W Robinson JP Rothman M Royal MA Simon L Stauffer JW Stein W Tollett J Wernicke J Witter J; IMMPACT. Core outcome measures for chronic pain clinical trials: IMMPACT recommendations. PAIN 2005;113:9–19. 15621359 10.1016/j.pain.2004.09.012

[43] Dworkin RH Turk DC Katz NP Rowbotham MC Peirce-Sandner S Cerny I Clingman CS Eloff BC Farrar JT Kamp C McDermott MP Rappaport BA Sanhai WR Evidence-based clinical trial design for chronic pain pharmacotherapy: a blueprint for ACTION. PAIN 2011;152(3 suppl):S107–115. 21145657 10.1016/j.pain.2010.11.008

[44] Dworkin RH Turk DC Peirce-Sandner S Baron R Bellamy N Burke LB Chappell A Chartier K Cleeland CS Costello A Cowan P Dimitrova R Ellenberg S Farrar JT French JA Gilron I Hertz S Jadad AR Jay GW Kalliomaki J Katz NP Kerns RD Manning DC McDermott MP McGrath PJ Narayana A Porter L Quessy S Rappaport BA Rauschkolb C Reeve BB Rhodes T Sampaio C Simpson DM Stauffer JW Stucki G Tobias J White RE Witter J Research design considerations for confirmatory chronic pain clinical trials: IMMPACT recommendations. PAIN 2010;149:177–93. 20207481 10.1016/j.pain.2010.02.018

[45] Egsmose C Lund B Bach Andersen R Hip joint distension in osteoarthrosis. A triple-blind controlled study comparing the effect of intra-articular indoprofen with placebo. Scand J Rheumatol 1984;13:238–42. 6385228 10.3109/03009748409100392

[46] Enck P Bingel U Schedlowski M Rief W The placebo response in medicine: minimize, maximize or personalize? Nat Rev Drug Discov 2013;12:191–204. 23449306 10.1038/nrd3923

[47] Enck P Klosterhalfen S Weimer K Horing B Zipfel S The placebo response in clinical trials: more questions than answers. Philos Trans R Soc Lond B Biol Sci 2011;366:1889–95. 21576146 10.1098/rstb.2010.0384 PMC3130397

[48] Falco FJ Manchikanti L Datta S Sehgal N Geffert S Onyewu CO Singh V Bryce DA Benyamin R Simopoulos TT Vellejo R Gupta S Ward SP Hirsch JA An update of the systematic assessment of the diagnostic accuracy of lumbar facet joint nerve blocks. Pain Physician 2012;15:E869–907. 23159979

[49] Fejer R Kyvik KO Hartvigsen J The prevalence of neck pain in the world population: a systematic critical review of the literature. Eur Spine J 2006;15:834–48. 15999284 10.1007/s00586-004-0864-4 PMC3489448

[50] Flacco ME Manzoli L Boccia S Capasso L Aleksovska K Rosso A Scaioli G De Vito C Siliquini R Villari P Ioannidis JP Head-to-head randomized trials are mostly industry sponsored and almost always favor the industry sponsor. J Clin Epidemiol 2015;68:811–20. 25748073 10.1016/j.jclinepi.2014.12.016

[51] Flacco ME Manzoli L Ioannidis JP Noninferiority is almost certain with lenient noninferiority margins. J Clin Epidemiol 2016;71:118. 10.1016/j.jclinepi.2015.11.010 26607237

[52] Freburger JK Holmes GM Agans RP Jackman AM Darter JD Wallace AS Castel LD Kalsbeek WD Carey TS The rising prevalence of chronic low back pain. Arch Intern Med 2009;169:251–8. 19204216 10.1001/archinternmed.2008.543 PMC4339077

[53] Freeman BJ Fraser RD Cain CM Hall DJ Chapple DC A randomized, double-blind, controlled trial: intradiscal electrothermal therapy versus placebo for the treatment of chronic discogenic low back pain. Spine (Phila Pa 1976) 2005;30:2369–77; discussion 2378. 16261111 10.1097/01.brs.0000186587.43373.f2

[54] Friedly J Chan L Deyo R Geographic variation in epidural steroid injection use in medicare patients. J Bone Joint Surg Am 2008;90:1730–7. 18676905 10.2106/JBJS.G.00858 PMC2657306

[55] Friedly JL Comstock BA Turner JA Heagerty PJ Deyo RA Sullivan SD Bauer Z Bresnahan BW Avins AL Nedeljkovic SS Nerenz DR Standaert C Kessler L Akuthota V Annaswamy T Chen A Diehn F Firtch W Gerges FJ Gilligan C Goldberg H Kennedy DJ Mandel S Tyburski M Sanders W Sibell D Smuck M Wasan A Won L Jarvik JG A randomized trial of epidural glucocorticoid injections for spinal stenosis. N Engl J Med 2014;371:11–21. 24988555 10.1056/NEJMoa1313265

[56] Geurts JW van Wijk RM Stolker RJ Groen GJ Efficacy of radiofrequency procedures for the treatment of spinal pain: a systematic review of randomized clinical trials. Reg Anesth Pain Med 2001;26:394–400. 11561257 10.1053/rapm.2001.23673

[57] Ghahreman A Ferch R Bogduk N The efficacy of transforaminal injection of steroids for the treatment of lumbar radicular pain. Pain Med 2010;11:1149–68. 20704666 10.1111/j.1526-4637.2010.00908.x

[58] Higgins JT Altman DG Sterne JC The Cochrane Collaboration's tool for assessing risk of bias in randomised trials. BMJ 2011;343:d5928. 22008217 10.1136/bmj.d5928 PMC3196245

[59] Horng S Miller FG Is placebo surgery unethical? N Engl J Med 2002;347:137–9. 12110744 10.1056/NEJMsb021025

[60] Hrobjartsson A Thomsen AS Emanuelsson F Tendal B Hilden J Boutron I Ravaud P Brorson S Observer bias in randomised clinical trials with binary outcomes: systematic review of trials with both blinded and non-blinded outcome assessors. BMJ 2012;344:e1119. 22371859 10.1136/bmj.e1119

[61] Jacobs WC Rubinstein SM Willems PC Moojen WA Pellise F Oner CF Peul WC van Tulder MW The evidence on surgical interventions for low back disorders, an overview of systematic reviews. Eur Spine J 2013;22:1936–49. 23681497 10.1007/s00586-013-2823-4 PMC3777049

[62] Jensen KB Kaptchuk TJ Chen X Kirsch I Ingvar M Gollub RL Kong J A neural mechanism for nonconscious activation of conditioned placebo and nocebo responses. Cereb Cortex 2015;25:3903–10. 25452576 10.1093/cercor/bhu275 PMC4585522

[63] Jonas WB Crawford C Colloca L Kaptchuk TJ Moseley B Miller FG Kriston L Linde K Meissner K To what extent are surgery and invasive procedures effective beyond a placebo response? A systematic review with meta-analysis of randomised, sham controlled trials. BMJ Open 2015;5:e009655. 10.1136/bmjopen-2015-009655 PMC4679929 26656986

[64] Kallmes DF Comstock BA Heagerty PJ Turner JA Wilson DJ Diamond TH Edwards R Gray LA Stout L Owen S Hollingworth W Ghdoke B Annesley-Williams DJ Ralston SH Jarvik JG A randomized trial of vertebroplasty for osteoporotic spinal fractures. N Engl J Med 2009;361:569–79. 19657122 10.1056/NEJMoa0900563 PMC2930487

[65] Kaptchuk TJ Goldman P Stone DA Stason WB Do medical devices have enhanced placebo effects? J Clin Epidemiol 2000;53:786–92. 10942860 10.1016/s0895-4356(00)00206-7

[66] Kaptchuk TJ Miller FG Placebo effects in medicine. N Engl J Med 2015;373:8–9. 26132938 10.1056/NEJMp1504023

[67] Kaptchuk TJ Stason WB Davis RB Legedza AR Schnyer RN Kerr CE Stone DA Nam BH Kirsch I Goldman RH Sham device v inert pill: randomised controlled trial of two placebo treatments. BMJ 2006;332:391–7. 16452103 10.1136/bmj.38726.603310.55 PMC1370970

[68] Kapural L Vrooman B Sarwar S Krizanac-Bengez L Rauck R Gilmore C North J Girgis G Mekhail N A randomized, placebo-controlled trial of transdiscal radiofrequency, biacuplasty for treatment of discogenic lower back pain. Pain Med 2013;14:362–73. 23279658 10.1111/pme.12023

[69] Kapural L Yu C Doust MW Gliner BE Vallejo R Sitzman BT Amirdelfan K Morgan DM Brown LL Yearwood TL Bundschu R Burton AW Yang T Benyamin R Burgher AH Novel 10-kHz high-frequency therapy (HF10 therapy) is superior to traditional low-frequency spinal cord stimulation for the treatment of chronic back and leg pain: the SENZA-RCT randomized controlled trial. Anesthesiology 2015;123:851–60. 26218762 10.1097/ALN.0000000000000774

[70] Kennedy DJ Engel A Kreiner DS Nampiaparampil D Duszynski B MacVicar J Fluoroscopically guided diagnostic and therapeutic intra-articular sacroiliac joint injections: a systematic review. Pain Med 2015;16:1500–18. 26178855 10.1111/pme.12833

[71] Kim KS Yoon ST Li J Park JS Hutton WC Disc degeneration in the rabbit: a biochemical and radiological comparison between four disc injury models. Spine (Phila Pa 1976) 2005;30:33–7. 15626978 10.1097/01.brs.0000149191.02304.9b

[72] King W Ahmed SU Baisden J Patel N Kennedy DJ Duszynski B MacVicar J Diagnosis and treatment of posterior sacroiliac complex pain: a systematic review with comprehensive analysis of the published data. Pain Med 2015;16:257–65. 25677327 10.1111/pme.12630

[73] Kirkley A Birmingham TB Litchfield RB Giffin JR Willits KR Wong CJ Feagan BG Donner A Griffin SH D'Ascanio LM Pope JE Fowler PJ A randomized trial of arthroscopic surgery for osteoarthritis of the knee. N Engl J Med 2008;359:1097–107. 18784099 10.1056/NEJMoa0708333

[74] Kong J Spaeth R Cook A Kirsch I Claggett B Vangel M Gollub RL Smoller JW Kaptchuk TJ Are all placebo effects equal? Placebo pills, sham acupuncture, cue conditioning and their association. PLoS One 2013;8:e67485. 23935833 10.1371/journal.pone.0067485 PMC3729687

[75] Koshi EB Short CA Placebo theory and its implications for research and clinical practice: a review of the recent literature. Pain Pract 2007;7:4–20. 17305673 10.1111/j.1533-2500.2007.00104.x

[76] Kumar K Taylor RS Jacques L Eldabe S Meglio M Molet J Thomson S O'Callaghan J Eisenberg E Milbouw G Buchser E Fortini G Richardson J North RB Spinal cord stimulation versus conventional medical management for neuropathic pain: a multicentre randomised controlled trial in patients with failed back surgery syndrome. PAIN 2007;132:179–88. 17845835 10.1016/j.pain.2007.07.028

[77] Li S Qi M Yuan W Chen H The impact of the depression and anxiety on prognosis of cervical total disc replacement. Spine (Phila Pa 1976) 2015;40:E266–271. 25494313 10.1097/BRS.0000000000000743

[78] Maas ET Ostelo RW Niemisto L Jousimaa J Hurri H Malmivaara A van Tulder MW Radiofrequency denervation for chronic low back pain. Cochrane Database Syst Rev 2015;CD008572. 26495910 10.1002/14651858.CD008572.pub2 PMC8782593

[79] Manchikanti L Hirsch JA Cohen SP Heavner JE Falco FJ Diwan S Boswell MV Candido KD Onyewu CO Zhu J Sehgal N Kaye AD Benyamin RM Helm S II Singh V Datta S Abdi S Christo PJ Hameed H Hameed M Vallejo R Pampati V Racz GB Raj PP Assessment of methodologic quality of randomized trials of interventional techniques: development of an interventional pain management specific instrument. Pain Physician 2014;17:E263–290. 24850111

[80] Manchikanti L Hirsch JA Heavner JE Cohen SP Benyamin RM Sehgal N Falco FJ Vallejo R Onyewu O Zhu J Kaye AD Boswell MV Helm S II Candido KD Diwan S Simopoulos TT Singh V Pampati V Racz GB Raj PP Development of an interventional pain management specific instrument for methodologic quality assessment of nonrandomized studies of interventional techniques. Pain Physician 2014;17:E291–317. 24850112

[81] Manchikanti L Pampati V Falco FJ Hirsch JA Growth of spinal interventional pain management techniques: analysis of utilization trends and Medicare expenditures 2000 to 2008. Spine (Phila Pa 1976) 2013;38:157–68. 22781007 10.1097/BRS.0b013e318267f463

[82] Manchikanti L Singh V Falco FJ Cash KA Fellows B Comparative outcomes of a 2-year follow-up of cervical medial branch blocks in management of chronic neck pain: a randomized, double-blind controlled trial. Pain Physician 2010;13:437–50. 20859313

[83] Manchikanti L Singh V Falco FJ Cash KA Pampati V Evaluation of the effectiveness of lumbar interlaminar epidural injections in managing chronic pain of lumbar disc herniation or radiculitis: a randomized, double-blind, controlled trial. Pain Physician 2010;13:343–55. 20648203

[84] Margetic P Pavic R Stancic MF Provocative discography screening improves surgical outcome. Wien Klin Wochenschr 2013;125:600–10. 23989460 10.1007/s00508-013-0404-5

[85] Moseley JB O'Malley K Petersen NJ Menke TJ Brody BA Kuykendall DH Hollingsworth JC Ashton CM Wray NP A controlled trial of arthroscopic surgery for osteoarthritis of the knee. N Engl J Med 2002;347:81–8. 12110735 10.1056/NEJMoa013259

[86] North RB Kidd DH Farrokhi F Piantadosi SA Spinal cord stimulation versus repeated lumbosacral spine surgery for chronic pain: a randomized, controlled trial. Neurosurgery 2005;56:98–106; discussion 106–107. 10.1227/01.neu.0000144839.65524.e0 15617591

[87] North RB Kumar K Wallace MS Henderson JM Shipley J Hernandez J Mekel-Bobrov N Jaax KN Spinal cord stimulation versus re-operation in patients with failed back surgery syndrome: an international multicenter randomized controlled trial (EVIDENCE study). Neuromodulation 2011;14:330–5; discussion 335–336. 21992427 10.1111/j.1525-1403.2011.00371.x

[88] Pauza KJ Howell S Dreyfuss P Peloza JH Dawson K Bogduk N A randomized, placebo-controlled trial of intradiscal electrothermal therapy for the treatment of discogenic low back pain. Spine J 2004;4:27–35. 14749191 10.1016/j.spinee.2003.07.001

[89] Pearl J Causality: models, reasoning and inference. Cambridge, United Kingdom: Cambridge University Press, 2009.

[90] Petersen GL Finnerup NB Grosen K Pilegaard HK Tracey I Benedetti F Price DD Jensen TS Vase L Expectations and positive emotional feelings accompany reductions in ongoing and evoked neuropathic pain following placebo interventions. PAIN 2014;155:2687–98. 25281929 10.1016/j.pain.2014.09.036

[91] Platt R Takvorian SU Septimus E Hickok J Moody J Perlin J Jernigan JA Kleinman K Huang SS Cluster randomized trials in comparative effectiveness research: randomizing hospitals to test methods for prevention of healthcare-associated infections. Med Care 2010;48(6 suppl):S52–57. 20473200 10.1097/MLR.0b013e3181dbebcf

[92] Provenzano DA Lassila HC Somers D The effect of fluid injection on lesion size during radiofrequency treatment. Reg Anesth Pain Med 2010;35:338–42. 20607874 10.1097/aap.0b013e3181e82d44

[93] Rauck R Coffey RJ Schultz DM Wallace MS Webster LR McCarville SE Grigsby EJ Page LM Intrathecal gabapentin to treat chronic intractable noncancer pain. Anesthesiology 2013;119:675–86. 23835590 10.1097/ALN.0b013e3182a10fbf

[94] Rauck RL Wallace MS Leong MS Minehart M Webster LR Charapata SG Abraham JE Buffington DE Ellis D Kartzinel R; Ziconotide 301 Study G. A randomized, double-blind, placebo-controlled study of intrathecal ziconotide in adults with severe chronic pain. J Pain Symptom Manage 2006;31:393–406. 16716870 10.1016/j.jpainsymman.2005.10.003

[95] Rosen A Yi J Kirsch I Kaptchuk TJ Ingvar M Jensen KB Effects of subtle cognitive manipulations on placebo analgesia—an implicit priming study. Eur J Pain 2017;21:594–604. 27748563 10.1002/ejp.961 PMC5363385

[96] Ruan X Tran L Kaye AD Harnessing positive placebo effect and minimize negative nocebo effect: the art of a healing profession. PAIN 2016;157:2390. 10.1097/j.pain.0000000000000643 27643837

[97] Savovic J Jones HE Altman DG Harris RJ Juni P Pildal J Als-Nielsen B Balk EM Gluud C Gluud LL Ioannidis JP Schulz KF Beynon R Welton NJ Wood L Moher D Deeks JJ Sterne JA Influence of reported study design characteristics on intervention effect estimates from randomized, controlled trials. Ann Intern Med 2012;157:429–38. 22945832 10.7326/0003-4819-157-6-201209180-00537

[98] Schedlowski M Enck P Rief W Bingel U Neuro-bio-behavioral mechanisms of placebo and nocebo responses: implications for clinical trials and clinical practice. Pharmacol Rev 2015;67:697–730. 26126649 10.1124/pr.114.009423

[99] Sevel LS Craggs JG Price DD Staud R Robinson ME Placebo analgesia enhances descending pain-related effective connectivity: a dynamic causal modeling study of endogenous pain modulation. J Pain 2015;16:760–8. 26022539 10.1016/j.jpain.2015.05.001 PMC4522336

[100] Sevel LS O'Shea AM Letzen JE Craggs JG Price DD Robinson ME Effective connectivity predicts future placebo analgesic response: a dynamic causal modeling study of pain processing in healthy controls. Neuroimage 2015;110:87–94. 25659463 10.1016/j.neuroimage.2015.01.056 PMC4380552

[101] Smith GC Pell JP Parachute use to prevent death and major trauma related to gravitational challenge: systematic review of randomized controlled trials. BMJ 2003;327:1459–61. 14684649 10.1136/bmj.327.7429.1459 PMC300808

[102] Sox HC Goodman SN The methods of comparative effectiveness research. Annu Rev Public Health 2012;33:425–45. 22224891 10.1146/annurev-publhealth-031811-124610

[103] Sox HC Greenfield S Comparative effectiveness research: a report from the Institute of Medicine. Ann Intern Med 2009;151:203–5. 19567618 10.7326/0003-4819-151-3-200908040-00125

[104] Staats PS Yearwood T Charapata SG Presley RW Wallace MS Byas-Smith M Fisher R Bryce DA Mangieri EA Luther RR Mayo M McGuire D Ellis D Intrathecal ziconotide in the treatment of refractory pain in patients with cancer or AIDS: a randomized controlled trial. JAMA 2004;291:63–70. 14709577 10.1001/jama.291.1.63

[105] Stein N Sprenger C Scholz J Wiech K Bingel U White matter integrity of the descending pain modulatory system is associated with interindividual differences in placebo analgesia. PAIN 2012;153:2210–17. 22959599 10.1016/j.pain.2012.07.010

[106] Swerdlow M Sayle-Creer WS A study of extradural medication in the relief of the lumbosciatic syndrome. Anaesthesia 1970;25:341–5. 4193992 10.1111/j.1365-2044.1970.tb00218.x

[107] Tavel ME The placebo effect: the good, the bad, and the ugly. Am J Med 2014;127:484–8. 24518105 10.1016/j.amjmed.2014.02.002

[108] Tetreault P Mansour A Vachon-Presseau E Schnitzer TJ Apkarian AV Baliki MN Brain connectivity predicts placebo response across chronic pain clinical trials. PLoS Biol 2016;14:e1002570. 27788130 10.1371/journal.pbio.1002570 PMC5082893

[109] Tracey I Getting the pain you expect: mechanisms of placebo, nocebo and reappraisal effects in humans. Nat Med 2010;16:1277–83. 20948533 10.1038/nm.2229

[110] Tunis SR Stryer DB Clancy CM Practical clinical trials: increasing the value of clinical research for decision making in clinical and health policy. JAMA 2003;290:1624–32. 14506122 10.1001/jama.290.12.1624

[111] Turk DC Dworkin RH Allen RR Bellamy N Brandenburg N Carr DB Cleeland C Dionne R Farrar JT Galer BS Hewitt DJ Jadad AR Katz NP Kramer LD Manning DC McCormick CG McDermott MP McGrath P Quessy S Rappaport BA Robinson JP Royal MA Simon L Stauffer JW Stein W Tollett J Witter J Core outcome domains for chronic pain clinical trials: IMMPACT recommendations. PAIN 2003;106:337–45. 14659516 10.1016/j.pain.2003.08.001

[112] van Kleef M Barendse GA Kessels A Voets HM Weber WE de Lange S Randomized trial of radiofrequency lumbar facet denervation for chronic low back pain. Spine (Phila Pa 1976) 1999;24:1937–42. 10515020 10.1097/00007632-199909150-00013

[113] van Wijk RM Geurts JW Wynne HJ Hammink E Buskens E Lousberg R Knape JT Groen GJ Radiofrequency denervation of lumbar facet joints in the treatment of chronic low back pain: a randomized, double-blind, sham lesion-controlled trial. Clin J Pain 2005;21:335–44. 15951652 10.1097/01.ajp.0000120792.69705.c9

[114] Vase L Amanzio M Price DD Nocebo vs. placebo: the challenges of trial design in analgesia research. Clin Pharmacol Ther 2015;97:143–50. 25670519 10.1002/cpt.31

[115] Wallace MS Charapata SG Fisher R Byas-Smith M Staats PS Mayo M McGuire D Ellis D; Ziconotide Nonmalignant Pain Study G. Intrathecal ziconotide in the treatment of chronic nonmalignant pain: a randomized, double-blind, placebo-controlled clinical trial. Neuromodulation 2006;9:75–86. 22151630 10.1111/j.1525-1403.2006.00055.x

[116] Weinstein JN Tosteson TD Lurie JD Tosteson AN Blood E Hanscom B Herkowitz H Cammisa F Albert T Boden SD Hilibrand A Goldberg H Berven S An H Investigators S Surgical versus nonsurgical therapy for lumbar spinal stenosis. N Engl J Med 2008;358:794–810. 18287602 10.1056/NEJMoa0707136 PMC2576513

[117] Weinstein JN Tosteson TD Lurie JD Tosteson AN Hanscom B Skinner JS Abdu WA Hilibrand AS Boden SD Deyo RA Surgical vs nonoperative treatment for lumbar disk herniation: the Spine Patient Outcomes Research Trial (SPORT): a randomized trial. JAMA 2006;296:2441–50. 17119140 10.1001/jama.296.20.2441 PMC2553805

[118] Willems P Decision making in surgical treatment of chronic low back pain: the performance of prognostic tests to select patients for lumbar spinal fusion. Acta Orthop Suppl 2013;84:1–35. 10.3109/17453674.2012.753565 23427903

[119] Wood L Egger M Gluud LL Schulz KF Juni P Altman DG Gluud C Martin RM Wood AJ Sterne JA Empirical evidence of bias in treatment effect estimates in controlled trials with different interventions and outcomes: meta-epidemiological study. BMJ 2008;336:601–5. 18316340 10.1136/bmj.39465.451748.AD PMC2267990

